# The Effect of Enrichment on Leopard Geckos (*Eublepharis macularius*) Housed in Two Different Maintenance Systems (Rack System vs. Terrarium)

**DOI:** 10.3390/ani13061111

**Published:** 2023-03-21

**Authors:** Damian Zieliński

**Affiliations:** Department of Animal Ethology and Wildlife Management, University of Life Sciences in Lublin, Akademicka 13, 20-950 Lublin, Poland; damian.zielinski@up.lublin.pl; Tel.: +48-81-445-60-42

**Keywords:** environmental enrichment, leopard gecko, animal welfare, rack system, terrarium

## Abstract

**Simple Summary:**

Environmental enrichment is a method that improves animal welfare in captive conditions. There are many forms of enrichment, including olfactory, auditory, and structural enrichment, and their usefulness can be determined by measuring behavioral indicators. It is important to consider the behavioral needs of the animals for which the enrichment is intended when designing environmental enrichment. Therefore, the aim of this work was to test the utility of four types of environmental enrichment for leopard geckos kept in a terrarium or in a rack system.

**Abstract:**

The aim of this study was to test the usefulness of environmental enrichment for *Eublepharis macularius* depending on the maintenance method (terrarium vs. rack system). The hypothesis was that reptiles kept in an extremely low-stimulus environment (rack system) would be more prone to interact with environmental enrichment items than those kept in a biotope terrarium. During the study, 21 female geckos were kept in two types of captive enclosures: 9 in terrariums, and 12 in rack system boxes in groups of 3 animals per enclosure. During the enrichment sessions, geckos were observed for 45 min while enrichment items (dry and wet hides, a new feeding method, a new object) were present in the enclosure. All geckos showed interest in enrichment items that enabled hiding and climbing. Animals kept in the rack system showed significantly lower latency in approaching enrichment items and a higher frequency of enrichment interactions than lizards in biotope terrariums. However, no significant differences were found in the total time spent interacting with enrichment items between geckos in the two settings.

## 1. Introduction

Reptiles are popular pets around the world, although their welfare requirements in captivity are not always fulfilled, in part due to the apparent lack of awareness of their needs [1]. The management of exotic pets is challenging, given their biological needs (in comparison to companion animals), relatively poor adaptability to the captive environment, and the lack of availability of valuable and proven guidelines for the keeping and breeding of specific species of reptiles or amphibians [2,3]. Additionally, the situation is often aggravated by the lack of specialized veterinary care [4] and the lower inclination of owners to use such care [5,6,7]. Additionally, most owners are not fully compliant with care guidelines and lack a basic understanding of the needs of reptiles in captivity [3,4,8]. Modern media has a strong influence on the pet trade. The Internet provides an easy way to search for information about various exotic species, thus driving the demand for new, unusual pets [9,10]. However, the care guidelines available on various websites are often incorrect and misleading [11]. Even with the easy availability of valuable data about their needs, many reptiles are kept in inadequate conditions [1]. 

Pet reptiles can be kept in three main ways: in a biotope terrarium; in a rack system, i.e., a special system in which the animals are kept in plastic containers in a relatively limited space and without additional furnishing (limited stimuli); or in bigger plastic containers (e.g., Herptek), with or without enrichment items [2,12,13]. In the case of the mass breeding of reptiles, rack systems are the most common forms of maintenance, especially for lizards and small snakes, and Herptek is more commonly used for bigger species [12,13]. However, this type of maintenance involves significant space constraints, which can result in diminished animal welfare and reduced expression of species-specific behaviors [14,15]. 

Reptiles are known for a wide range of abnormal behaviors and health-related issues resulting from the captive environment [12]. Reptiles that lack appropriate environmental conditions (e.g., temperature and humidity, UVB lighting, hiding places, proper nutrition, and environmental enrichment) are more susceptible to diseases than those kept in appropriate conditions [16]. The enclosure type and size rarely resembles the animal’s normal home range. For example, the size of the natural habitat of *Elaphe guttata emoryi* covers an average area of 10.17 ha [17]. In contrast, the recommended enclosure size for this species kept in captivity covers 0.000024 ha [18]. This also does not cover the dimensions suggested by Warwick et al.’s [2] guideline and diameter formula for reptiles (which indicates that reptiles should have more space in the linear dimension than their length). Moreover, inappropriate environmental conditions and frequent manipulation in the terrarium (e.g., replacing substrate, moving cages, changing UVB lamps, adding accessories, adding new animals, handling, or placing the cage in a high-traffic area) cause severe stress [19].

Regarding the cognitive challenges for exotic animals, special attention should be paid to allow them the possibility to manifest their natural behaviors. To achieve this, it is necessary to provide the animals with a complex environment that resembles the natural biotope of the species, improved by adding numerous environmental enrichment items. Then captivity can follow the “life worth living” trend [20], and animals in captivity will have the opportunity to properly explore and inspect their environment and will be able to satisfy their species-specific behavioral needs [21]. Nevertheless, despite the proven usefulness of different environmental enhancements, it is equally important to work continuously on finding new solutions, evaluating existing ones, and creating new forms of enrichment (aiming for different types of behaviors) to be able to change the captive environment even daily, especially for animals kept in a low-stimulus rack system. Reptile breeders who use rack systems should focus more on attempting to mitigate the inevitable deprivation caused by this type of captive environment by using tested environmental enrichment items. 

Healthy behavior exhibited by a relaxed reptile include species-specific behaviors (e.g., foraging, thermoregulatory behavior, exploration) and behavioral diversity (unhurried body movements, regular resting habits, relaxed eating, use of the entire enclosure for locomotion) [22,23,24,25]. The environmental enrichment strategy aims to improve the welfare and enrich the behavioral diversity of animals in captivity [21,26,27,28]. The creation of a naturalistic environment, which, to a small extent, can resemble the natural habitat of the animal, promotes well-being and enables the animals to manifest species-specific behaviors [29]. It is true that it can be challenging to create a comprehensive replica of an animal’s natural habitat in captivity [30]. Using enrichment items, even in biotope tanks, and especially in rack systems, seems to improve the welfare of animals to a greater or lesser extent, and can increase their plasticity in captivity [31,32]. Furthermore, observing the animals’ reactions to the introduced enrichment and analyzing their behavior toward these stimuli can allow for the evaluation and further improvement of environmental enrichment items [33].

There is less research on environmental enrichment for reptiles than there is for other animal groups. Between 2002 and 2014, studies on this topic accounted for only 7% of all published work in this area (including reptiles and amphibians together) [33]. Research on the utility of enrichment items for reptiles is time-consuming due to the great diversity of this group, as well as the need to adapt to the behavioral needs of each species [22,29,34]. Experiments conducted so far on the use of environmental enrichment items for reptiles in captivity have shown that positive results are achieved with those that fit the behavioral needs of the species. For example, green anoles (*Anolis carolinensis*), five-lined skinks (*Plestiodon fasciatus*), and blue-tongued skinks (*Tiliqua scincoides*) displayed more foraging behavior when fed with live free-running insects than when insects were placed in the feeding bowl [35,36], and the use of feeder puzzles increased feeding time for fly river turtles (*Carettochelys insculpta*) [37]. However, studies on tree-runner lizards (*Plica plica*) using food enrichment showed no statistical differences in latency to first strike, duration of hunting activity, or type of enclosure in scatter and enriched trials [38]. The behavior of ball pythons (*Python regius*) was more diverse if they had access to an enriched environment in a biotope terrarium [13], and corn snakes (*Pantherophis guttatus*), when given a choice, showed a preference for a large enclosure [14]. Eastern box turtles (*Terrapene carolina carolina*) spent more time trying to escape and less time resting when kept in empty rooms than in enriched rooms [39]. Enrichment for eastern fence lizards (*Sceloporus undulatus*) in the form of raised basking platforms did not affect factors related to rates of behavior, physiological aspects, and stress hormone levels [40]. Leopard geckos (*Eublepharis mascularius*) responded mainly to two of the five environmental enrichment items tested, thermal and feeding, which meet the behavioral needs of this species [23]. 

Therefore, this study aimed to test the usefulness of four environmental enrichment items that allowed for climbing, hiding, foraging, and playing in different maintenance environments (terrarium vs. rack system) for *E. macularius*. The hypothesis was that reptiles kept in an extremely low-stimulus environment (rack system) would be more prone to interact with environmental enrichment items than those kept in a biotope terrarium. 

## 2. Materials and Methods

### 2.1. Animals and Enclosures

**Figure 1:**
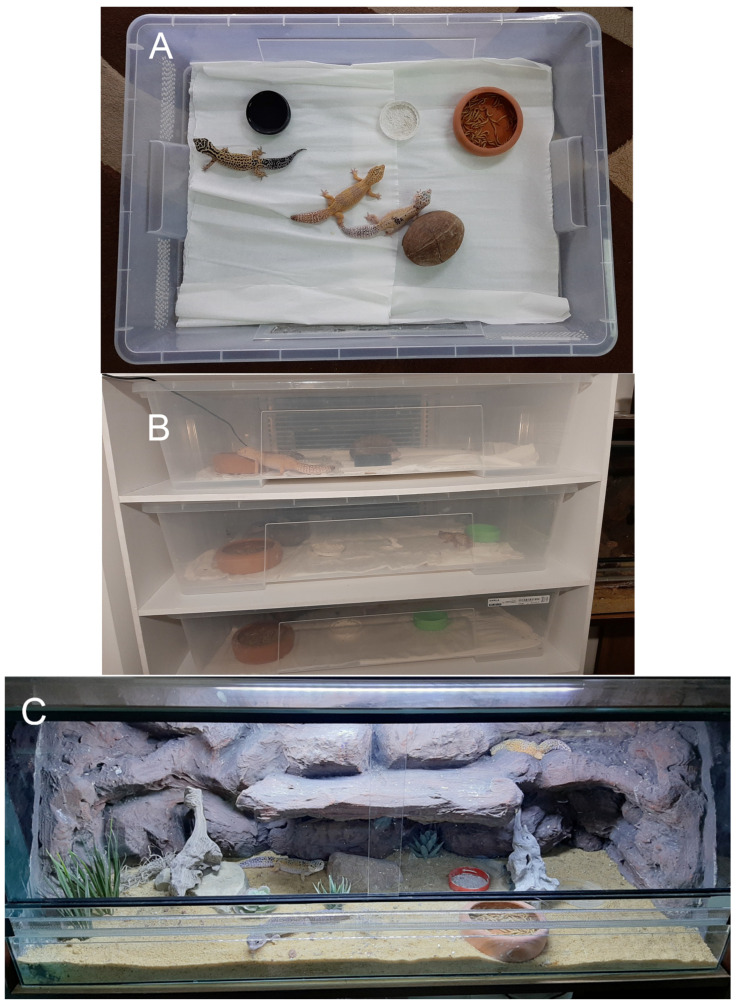
Captive environments used in the study. (**A**,**B**) rack system, (**C**) biotope terrarium.

For this study, 21 adult female leopard geckos (*E. macularius*) were used. The lizards were approximately 2 years of age, and were obtained from Tropical Factory (Kalisz, Poland). They were randomly divided into two groups: 9 females in terrariums and 12 females in a rack system (Figure 1), and randomly divided into particular boxes or terrariums. In both groups, 3 females were placed in each enclosure. They were kept in groups because in captive conditions, leopard geckos commonly gather in small groups, including several females (up to 10) and one dominant male [41]. Only females were used in this study because the courtship behavior in this species is related to hierarchy and could affect the behavioral response to enrichment, as social experience affects territorial and reproductive behavior [42]. After the study, the animals were sold.

Three glass biotope terrariums (100 cm × 40 cm × 40 cm—length × width × height— Tropical Factory, Kalisz, Poland) were used, in which natural conditions (basic level of enriched environment) were reproduced by building a rear wall, with artificial rocks and numerous shelves and hiding places (Figure 1C). The ground was covered with a substrate on which natural stones and artificial succulent plants were placed. The second group was maintained in a rack system (Tropical Factory, Kalisz, Poland) as an extremely low enrichment environment. In this system, three females were placed in each plastic box (78 cm × 56 cm × 18 cm—length × width × height—SAMLA box, Ikea, Poland) with kitchen paper towels on the bottom and one hide (half of a coconut shell). The boxes were stacked one above the other in the rack system (Figure 1A,B). The terrariums and rack boxes were equipped with bowls for food, water, and a calcium source. Each box in the rack system and each terrarium was illuminated with LED light (from above) and heated with a heating mat placed under the terrarium or on the back of the rack (rack system). The temperature during the day was 28 °C, and the relative humidity was 40%. All enclosures were stacked next to each other in the same room. The terrariums were placed one on top of the other in a storage unit with shelves. Animals in individual tanks could not see the animals in other tanks. All daily routines, as well as test procedures, were performed randomly.

Each enclosure with reptiles was maintained at a 12 h day/night cycle, with lights on at 08:00 and off at 20:00. The animals were fed with crickets (*Gryllodes sigillatus*), mealworms (*Tenebrio molitor*), and superworms (*Zoophobas morio*) 3 times per week (feeder insects obtained from Bugstore, Kraków, Poland), with constant access to water and calcium. They were handled once a week during enclosure cleaning. The terrariums used in the study met the suggested absolute minimum requirements for reptiles set forth by Warwick et al. [2], while the containers in the rack system did not meet the requirements in terms of size; environmental conditions (temperature, humidity, food, access to water) were met, but lacked an adequate level of environmental enrichment.

### 2.2. Environmental Enrichment Items

As mentioned above, four types of environmental enrichment were used in this study. During each enrichment session (trial), the enrichment items were placed in the enclosure (terrarium or rack box). In the dry hide enrichment session, cork oak (*Quercus suber*) bark was used, which served as both a hide (cave) and a climbing element for the animals. This type of enrichment is a popular terrarium decoration that is available in pet stores in most countries. In the wet hide enrichment session, the animals were presented with a plastic container filled with a moist substrate with a hole in the lid as an entrance. This kind of enrichment fulfills the natural behavior of lizards, especially during shedding, and for females, it provides the possibility to dig in the ground during the breeding season when they are looking for a place to lay eggs. The third enrichment was related to a new way of feeding, using a translucent plastic container (a urine test container) in which several holes were drilled. Several feeder insects of different sizes were placed in the container: some were able to escape from the container and some remained inside. The last type of enrichment was a new object in the form of a rubber dog toy (Trixie, Tarp, Germany). The last enrichment was not related to any specific behavioral needs of the geckos; it was used to test whether the addition of any object to the animals’ environment would cause a response in the form of interaction with the object.

### 2.3. Enrichment Scheme

The reptiles were maintained for 13 weeks in baseline conditions, without any additional enrichment items, which was a period of acclimatization to their environment (terrarium or rack system). Next, enrichment items were used for observations in 2 sessions (repetitions). In this study, 4 types of environmental enrichment were used: a dry hide, a wet hide, a new feeding method, and a new object. The enrichment sessions (trials) were performed in the evening when the lighting and heating were switched off (between 20:00 and 24:00). The enrichment item was placed in the enclosure for 45 min and then removed, and this was repeated the same way for the next 2 nights (each enrichment was used 3 times in 1 session). In the trial, 1 enrichment item was used in each tank for 3 individuals. It was placed mostly in a central location on the bottom of the enclosure (also on the sides, in the case of wet hide in the terrarium, due to the lack of space in the center for this size of enrichment). There was a 24 h interval between the last repetition of one type of enrichment and the first of the next type. The geckos’ behavior during the sessions was recorded with a video camera (Sony, HDR-CX405, Sony Europe B.V., Surrey, UK). After the first enrichment session, there was a 7 day break, with no additional enrichment items, then the enrichment scheme was repeated. In total, each enrichment type was used 6 times in every enclosure. The recordings from the enrichment sessions were analyzed. The recordings were analyzed frame by frame using CyberLink PowerDirector (version 18.0.2402.0; CyberLink Corp., New Taipei City, Taiwan). A separate analysis was performed for each gecko from each terrarium/container.

### 2.4. Statistical Analysis

Statistical analysis of the study results was carried out using the Statistica 13.3 statistical package. The basic classical statistical measures of the arithmetic mean (M) and standard deviation (SD) were used to describe the distributions of the studied characteristics. For traits with distributions that differed significantly from the normal distribution, the statistical description was supplemented by a positional measure of the average value, the median (Me). To examine the differences in lizards’ interest in the studied enrichment types according to the environment (terrarium vs. rack) and enrichment item (dry hide, feeding, new object, wet hide), the analysis of variance (ANOVA), with repeated measures, and Tukey’s test for pairwise comparison of means were used. The conformity of the distributions of the studied traits to the normal distribution was assessed using the Shapiro–Wilk test. The results were considered statistically significant at the typical level of *p* ≤ 0.05. 

The experimental units in this study were each enclosure with 3 geckos during a 45 min trial with environmental enrichment. In determining the level of interaction with the enrichment items, averaged values based on the behavior of all animals in a given tank were considered. The N values used in the study were N = 3 for the terrarium environment (with 9 geckos) and N = 4 for the rack system environment (with 12 geckos).

## 3. Results

**Figure 2:**
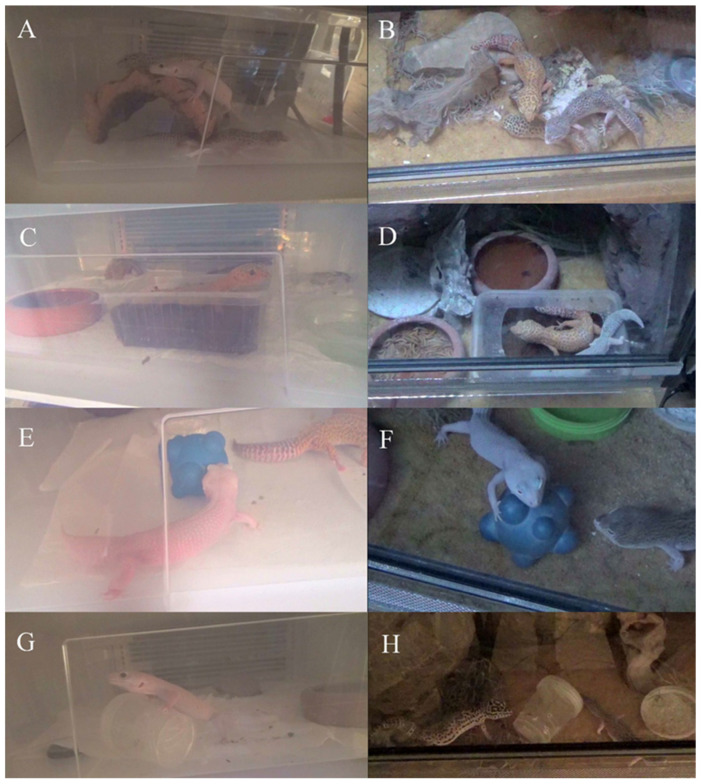
Environmental enrichment items used in the study: (**A**,**B**) dry hide; (**C**,**D**) wet hide; (**E**,**F**) new object; (**G**,**H**) feeding. (**A**,**C**,**E**,**F**) Rack system; (**B**,**D**,**F**,**H**) biotope terrarium.

The types of gecko behavior toward the given enrichment were determined based on the analysis of video films from the enrichment sessions (Table S1). Four main state behaviors (observation, resting on/in/under, circling the enrichment, climbing) and six occasional (event) behaviors (licking, sniffing, scratching, digging, hunting, manipulation) that occurred during the enrichment sessions were observed. The involvement of each individual interaction in the enrichment session was determined in detail by counting the number of single contacts with the enrichment item (touch) and measuring the duration of the contact. The exact extent of each lizards’ interest in the enrichment introduced into the environment is shown in Table 1, including the parameters studied: latency, number of interactions with the object, and total interaction time. The enrichment items used in the study engaged the geckos during the observed time. The lizards showed different degrees of interest in the objects placed in their enclosures (Figure 2).

### 3.1. Latency in Approaching Enrichment

**Figure 3:**
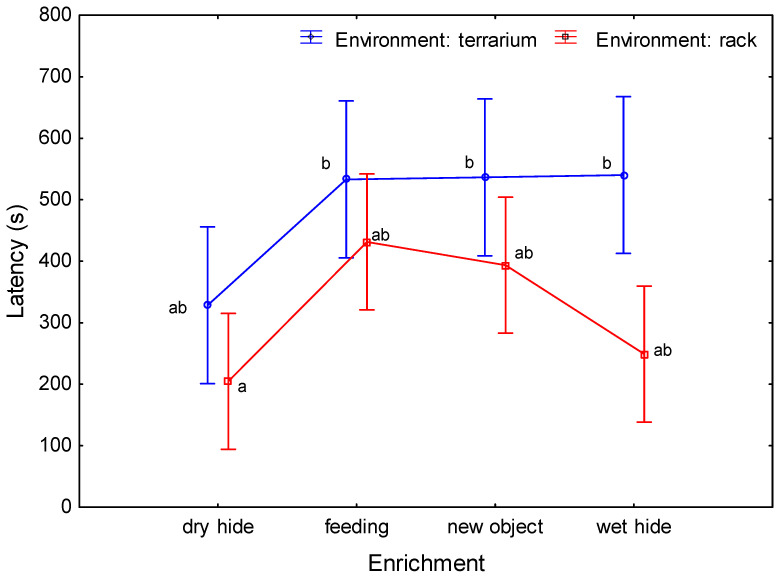
Medium latency (s) in approaching enrichment for studied environments (terrarium and rack) and enrichment items (vertical bars denote 0.95 confidence intervals; groups that do not have a common letter differ significantly).

Latency can be defined as the time from the start of the trial (placement of the enrichment item in the animal environment) to the lizard’s first contact with the item (touch, lick). Consistent with previous assumptions, analysis of variance showed significantly shorter latency in approaching the enrichment in the rack environment compared to the terrarium environment (319.7 ± 175.2 s and 484.6 ± 224.7 s, respectively; repeated measures ANOVA, F(1,20) = 16.605, *p* = 0.0006, $\eta_{p}^{2}$ = 0.45). In addition, significant differences were found in latency by enrichment type (repeated measures ANOVA, F(3,20) = 5.877, *p* = 0.005, $\eta_{p}^{2}$ = 0.47). The time to reach the dry hide enrichment was significantly shorter than the time to reach the feeding or new object enrichments. No significant differences were found in the time to reach wet hide and all other enrichment items or the time to reach feeding and new object enrichments. The time to reach the dry hide was significantly shorter in the rack environment than in the terrarium environment for all enrichment items, excluding the dry hide. No significant differences were found in the time to reach the dry hide in the terrarium compared to the other latencies, or in the time to reach enrichment in the rack (feeding, new object, and wet hide) compared to the other latencies (indicated with letters in Figure 3). No significant differences were detected between repetitions (repeated measures ANOVA, F(1,20) = 1.254, *p* = 0.276, $\eta_{p}^{2}$ = 0.06). 

### 3.2. Number of Interactions with Enrichment

The analysis of variance with repetitions made it possible to test for the presence of significant differences in the number of enrichment interactions, depending on the type of environment and type of enrichment. No significant differences were detected using analysis of variance with repetitions of sessions (repeated measures ANOVA, F(1,20) = 4.232, *p* = 0.053, $\eta_{p}^{2}$ = 0.17). However, the analysis of variance showed a significantly higher number of interactions with the enrichment in the rack environment compared to the terrarium environment (4.52 ± 2.65 and 3.11 ± 1.37 s, respectively; repeated measures ANOVA, F(1,20) = 9.585, *p* = 0.006, $\eta_{p}^{2}=$ 0.32). In addition, significant differences were found in the number of interactions with the enrichment, depending on the type of enrichment (repeated measures ANOVA, F(3,20) = 13.595, *p* = 0.00005, $\eta_{p}^{2}=$ 0.67). The highest number of interactions was observed with the dry hide enrichment. In addition, the number of interactions with the feeding enrichment was significantly higher compared to the number of interactions with the new object. No significant differences were found in the number of interactions with the feeding and wet hide enrichments. 

**Figure 4:**
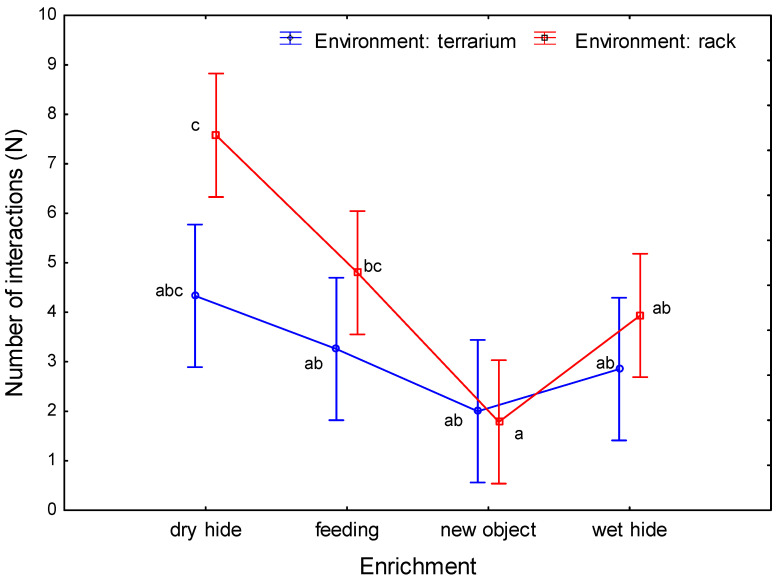
Average number of interactions (N) with enrichment for tested environments (terrarium, rack) and enrichment items (vertical bars denote 0.95 confidence intervals; groups that do not share a common letter differ significantly).

No significant interaction was detected for the two factors tested (repeated measures ANOVA, F(3,20) = 2.449, *p* = 0.093, $\eta_{p}^{2}$ = 0.27; Figure 4). With *p* < 0.1, some tendency for an ascending interaction can be noted, involving more significant disparities in the number of interactions in the rack environment compared to the terrarium environment. In addition, in the rack system, the number of interactions with the dry hide was significantly higher than the number of interactions with the new object and wet hide and significantly higher than the number of interactions in the terrarium environment with all other enrichment items, except the dry hide. In the rack system boxes, the lizards approached the new object significantly less often than the feeding and dry hide enrichments (Figure 4).

When comparing the numbers of animal interactions with the enrichment, it is notable that for each type of enrichment, the lizards in the rack system interacted with new objects in their environment more frequently than did those in the biotope terrarium (except for the new object).

### 3.3. Total Time Engaged with Enrichment

**Figure 5:**
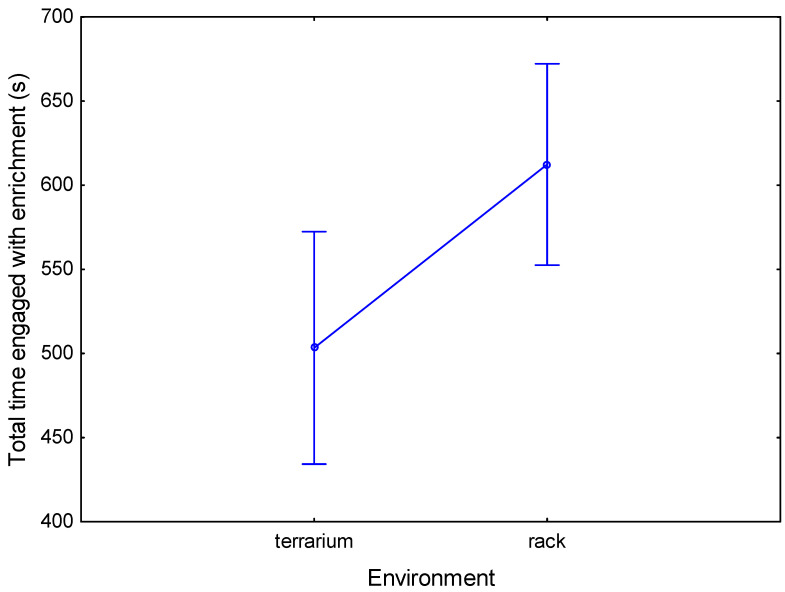
Mean total contact time (s) with the enrichment in both environments, without dividing by the enrichment type (vertical bars denote 0.95 confidence intervals, Tmax = 2700 s).

The last parameter analyzed was the total time of physical contact with the new object. This expresses the involvement with and level of interest of the lizards in the introduced enrichment in their environment. The analysis of variance showed significantly longer total contact time with the enrichment in the rack environment compared to the terrarium environment (612.4 ± 498.3 and 503.3 ± 415.3 s, respectively; repeated measures ANOVA, F(1,20) = 6.193; *p* = 0.022, $\eta_{p}^{2}$ = 0.24; Figure 5). 

**Figure 6:**
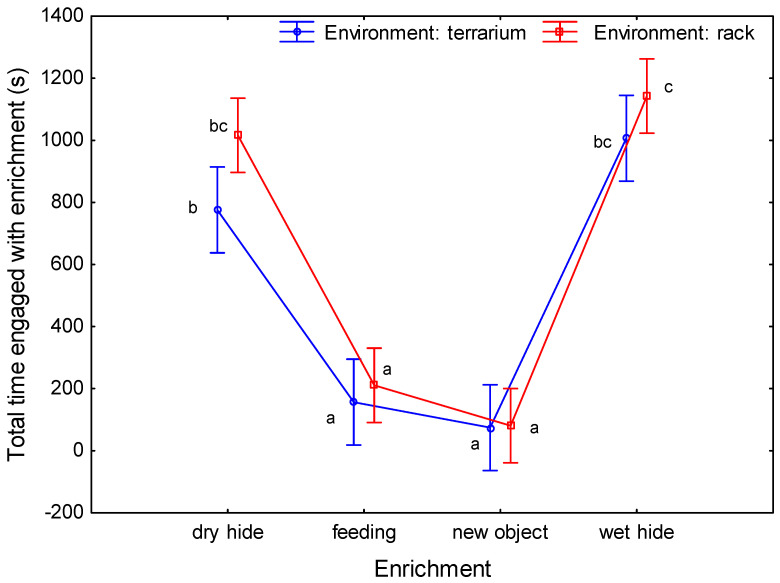
Mean total contact time (s) with enrichment items for tested environments (vertical bars denote 0.95 confidence intervals; groups that do not have a common letter differ significantly).

No significant differences were detected between repetitions (repeated measures ANOVA, F(1,20) = 2.122, *p* = 0.161, $\eta_{p}^{2}$ = 0.10). The total contact time with the wet and dry hides was significantly longer than with the feeding and new object enrichment items in both studied environments. No significant difference could be found in contact time between the wet hide and the dry hide in both environments. The total contact time with the new object and feeding enrichment items was significantly shorter than the contact time with other enrichment items in both environments (Figure 6). Notably, the lizards in both environments showed similar interest in the enrichment items (the line in the graph for both environments maintains the same trend), with almost identical total contact time with the new object (74.3 ± 37.2 s in the terrarium, 80.2 ± 69.9 s in the rack) and the most significant difference for the dry hide enrichment (775.6 ± 154.1 s in the terrarium, 1016.1 ± 185.4 s in the rack). Furthermore, lizards kept in the low-stimulus environment spent more time interacting with enrichment items than those in the terrarium, regardless of the type of enrichment. However, both groups of lizards were involved in the longest interactions with two types of enrichment, the dry and wet hide.

## 4. Discussion

The leopard geckos observed during the enrichment sessions engaged with all four types of environmental enrichment. However, their interest in interacting with the enrichment items varied due to specific species-oriented behaviors, which meant they performed the behavior differently (see Table S1) [33,43]. Most of the observed behaviors were related to the basic behavioral needs of the species; hence, the animals spent a significantly longer time with the hiding and climbing enrichment items (the wet and dry hides) than with the feeding or new object enrichments. Consistent with previous assumptions, the results showed that geckos kept in a rack system interacted more readily (latency), more often (frequency), and for a longer time (duration of total contact time) with environmental enrichment items compared to geckos kept in biotope terrariums. Similarly, when comparing these parameters, depending on the enrichment used during the session, geckos interacted more frequently and for more extended periods with enrichment items providing wet and dry hiding places. It is worth noting, however, that geckos in both environments responded to the enrichment in similar ways, differing only in the level of their response. Animals in the low-stimulus rack system responded to enrichment more quickly and for a longer time and stayed in contact with it more often during the observed time. The shorter latency shows that the rack system lizards were more motivated to interact with a new object in their living space.

Although the females were placed in rack systems, they did not habituate to conditions with reduced stimulus levels; instead, they continued to respond to enrichment, indicating a genetically determined need for environmental and behavioral differentiation. An enriched environment (e.g., complex biotope terrarium) improves reptiles’ cognitive performance and significantly improves their well-being [16,29]. The complexity of living conditions, including the way of offering food (dispersed release of food insects rather than in a bowl or feeder), increases the difficulty of locating and catching prey. Providing stimuli through diversifying the environment and inducing more hunting behavior in lizards significantly affects their activity level and natural behavior [38,44]. 

Environmental enrichments should be suitable for the species for which they will be used. The use of enrichment items not only improves the welfare of animals in captivity, but also affects specific aspects related to breeding (depending on its purpose), including reproductive success, foraging success, body condition, chances for successful reintroduction, or living in good physical and mental condition as a pet animal [14,45]. In this study, arbitrary enrichment (a dog toy as a new object) did not elicit significant responses from the animals, in contrast to enrichment items that targeted the specific exploration needs of geckos (such as a chance to climb and hide). It should also be remembered that additional elements in the existing environment provide the animals with new opportunities for shelter, thermoregulation, climbing, or rubbing, or aid in shedding. These features are extremely valuable, especially for animals living in a stimulus-poor environment such as a rack system, although Michaels et al. [45] pointed out that elements of basic equipment should not be considered as enrichment of the environment. However, in an equipment-poor environment in which lizards and snakes are most often kept in mass breeding conditions, adding even a “basic” element significantly enriches that environment. 

The observations of this study show that even leopard geckos in biotope terrariums responded to a routine change of substrate and a slight modification of the setting of existing equipment items with a significant change in behavior (increased exploration). I believe any change in the existing environment should be called enrichment, and temporary changes in equipment, including adding or relocation of items, within the environment should be included in basic husbandry. The search for new items to enrich the environment of animals in captivity should be constantly studied in different taxa. Surprisingly, the geckos tested in this study showed little interest in the new feeding method, in contrast to the results reported by Bashaw et al. [23], which showed that enriched feeding significantly increased exploration, foraging, and behavioral diversity. The device used in a study by Januszczak et al. [38] allowed lizards to better observe moving insects than in this study. The use of a semi-transparent container meant that the insects in the feeder were not fully visible to the geckos, which might have influenced their interest in this enrichment compared to other types. Studies show that the effectiveness of nutritional enrichment does not always meet researchers’ expectations and varies depending on the species studied and their dietary type (omnivore, insectivore, or herbivore). In one study, there was no effect of the designed feeder for *Plica plica* on the duration of hunting [38], while in another study, *Carettochelys insculpta* turtles took longer to feed using a puzzle feeder [37]. The use of an automatic feeder caused a decrease in foraging behavior and an increase in stereotypic aggressive and same-sex behavior in McCord’s snake-necked turtles (*Chelodina mccordi*), and an increase in stereotypic behavior (swimming against the glass) in Vietnamese pond turtles (*Mauremys annamensis*) [46]. Studies on enrichment with new objects for reptiles are focused on Testudinata, for which this enrichment is the most beneficial [43,47,48,49,50]. 

In this study, of all the applied enrichment items, the new object (rubber dog toy) aroused the least interest in the lizards. Perhaps applying an additional stimulus, e.g., a scent on the new object, would increase their reaction [40,51,52]. It is worth mentioning that the containers in the rack system used in this study were much larger than typical containers used by mass breeders of this species. It is therefore to be expected that keeping lizards or snakes in a very limited space likely involves even greater welfare deprivation. While the addition of environmental enrichment can improve animal welfare, this does not mean that the needs of animals in rack systems are met because there are many spatial, behavioral, chemical, visual, social, behavioral, and other aspects that are wholly or partially hindered by rack systems. The results of this study indicate suggestions for leopard gecko keepers regarding the kind of enrichment they should place in the enclosure to provide opportunities for the geckos to exhibit species-specific behaviors (such as a dry hide, for climbing or hiding). Therefore, providing the opportunity to climb/hide could improve the welfare of this species in captivity, although this requires further research on measuring welfare, which has not been done so far.

Many studies have proved the effect of captive environment complexity on behavior, immune response, and environmental preferences [39,48,49,53]. In the case of female leopard geckos, many affiliative behaviors can be observed, and aggression can occur when a new individual is introduced to an existing group (personal observation). It can be assumed that there is a risk of competition for new objects in the environment, which could result in one individual spending more time than others in the group interacting with the enrichment. However, for species that live in social groups, studies have not shown differences in their interaction with the enrichment, depending on the number of items added to the living space [54]. Moreover, an individuals’ interest in the enrichment may make it more attractive to others in the group, as a social facilitation effect [55].

With environmental enrichment, agonistic behavior can increase, remain unchanged, or decrease, depending on the methods used to assess aggression, the species studied, and the environmental enrichment used [56,57,58]. Aggressive behavior in group-housed animals during enrichment can occur in species with a clear hierarchy [59]. In such situations, studies have shown that enrichment does not change the number of antagonistic behaviors if adequate enrichment is provided for all individuals in the group [54,55]. Environmental enrichment items are widely studied for many animals, especially those kept in a zoo or a limited captivity environment [16,60]. The interest in providing environmental enrichment for reptiles is growing [32,46,61,62]. However, the research is focused on specific species rather than all reptiles in the class Reptilia [60]. Recently, a Welfare Quality^®^ protocol was compiled for *Tiliqua adelaidensis* [29], and the authors proposed that it could be used as the basis for developing taxon-specific tools considering species-specific biology.

### 4.1. The Ethical Implications of Rack Systems

It should be emphasized that despite the greater interaction with enrichment in the rack system environment, this should not be considered an incentive to keep reptiles in such conditions. The rack system represents a method of maintenance that, despite all efforts, cannot meet reptiles’ biological or behavioral needs [13]. This applies both to the size of the containers and the low level of environmental enrichment, which in the long run, leads to sensory deprivation and reduces animal welfare [43,47]. Based on the results of this study, it should be noted that the geckos in the rack system were so desperate for sensory stimulation that their response to the introduction of enrichment was almost instantaneous, and the number of approaches to the enrichment had the character of almost stereotypical repetition (in the case of the dry hides, especially). Unfortunately, the observed high popularity of rack systems among breeders of leopard geckos (and other nocturnal lizards), ball pythons, or western hognose snakes, demands that scientists undertake research on this topic and demonstrate the ethical inappropriateness of this method of reptile maintenance. 

### 4.2. Limitation of the Study

It is worth emphasizing some limitations of this work. First, the number of animals used in the study was low (9 geckos in a terrarium and 12 in a rack system) due to limited space for large enclosures. Therefore, the results cannot be generalized to all species of animals maintained in a low-stimulus environment. In addition, keeping the lizards in groups of three, which is how they are most often kept in captivity, made it necessary to treat the enclosures as a test unit in the statistical analysis. Future studies should be carried out to investigate the usefulness of enrichment items for other species of reptiles kept in a captive environment (e.g., *P. regius*, *Heterodon nasicus*, *Correlophus ciliatus*). Second, the time of observation (45 min) was not enough to fully understand the relationship between the geckos and the enrichment items. It is possible that the response during that length of time could be understood as a reaction to a novel object, rather than to a long-term object. Third, another limitation of this work is that it does not consider other breeding parameters for this species, in terms of between-sex behavioral interactions and different life-history stages. Fourth, the measurements made using the given approach run the risk of some overinterpretation due to differences in the size of the enclosures used in the study. Animals in rack boxes had a shorter path to enrichment than did the terrarium lizards. Fifth, the minor differences obtained in the environments studied here could also be explained by the relatively small difference in space that was provided to the reptiles. Probably, if a much larger biotope terrarium were used, the differences in the response of animals to these enrichment items would be greater. 

Furthermore, to the author’s knowledge, this is one of the first studies on the usefulness of enrichment items for animals kept in a low-stimulus (rack system) environment, which is common in the mass breeding of reptiles. Hence, it is worth continuing. Moreover, evaluations of reptile preferences may be equally useful in determining their preferred stimuli, and future research could explore ways to increase the effectiveness of such stimuli to increase the range of options available for animals in captivity. In addition, breeders could use preference evaluations to determine the highly preferred characteristics of enrichment items (e.g., shape, size, color, smell, hardness, structure); this information could be used to develop new enrichment strategies for reptiles, including species-specific or preferred behavioral types.

However, it is essential to consider that animals in captivity may habituate to the provided environment [63,64]. Therefore, the environment provided for the animal should be continuously modified to avoid habituation [33,61].

## 5. Conclusions

The enrichment of the environment of animals kept under low-stimulus conditions (a rack system) led to greater interest in enrichment. However, it can be noted that animals in the enriched terrarium biotope environment also responded significantly to enrichment. Lizards maintained in a stimulus-poor rack environment showed significantly lower latency and a more significant amount of enrichment interactions than lizards in biotope terrariums. However, no significant differences were found in the total interaction time with the enrichment items in the studied maintenance systems. Although the lizards were interested in the enrichment items used in the study, it should be emphasized that under rack system conditions, they could not fully exhibit species-specific behavior because there was a significant reduction in the availability of various stimuli (e.g., visual, social, organoleptic, or choice opportunity). Geckos prefer to be in a wet hide more than a dry hide that allows for climbing. It should be noted that geckos showed significant interest in this type of enrichment compared to others (feeding and new object enrichment items). The results of this research can serve as a simple guide for leopard gecko breeders to improve the diversity of behavior and welfare of these animals.

## Figures and Tables

**Table 1:** Latency (s), number of interactions (N), and total time of engagement with enrichment (s) depending on environment and enrichment in both repetitions. M, mean; SD, standard deviation.

	Enrichment		Environment						All Environments and Repeats
			Terrarium (M ± SD)			Rack (M ± SD)			
			Repeat			Repeat			
			1	2	All Repeats	1	2	All Repeats	
		N of Trials in Repetition	3	3	6	3	3	6	12
Latency (s)	Dry hide		263.8 ± 45.1	392.9 ± 199.4	328.4 ± 147.4	157.6 ± 100.6	251.7 ± 149.4	204.7 ± 128.2	257.7 ± 145.7
	Feeding		556.9 ± 257.5	509.4 ± 395.3	533.1 ± 299.5	455.5 ± 98.7	407.6 ± 119.3	431.5 ± 104.6	475.1 ± 207.7
	New object		313.5 ± 60.3	759.4 ± 132.8	536.5 ± 261.1	329.0 ± 73.6	458.4 ± 248.6	393.7 ± 183.3	454.9 ± 222.9
	Wet hide		595.0 ± 81.8	485.4 ± 135.8	540.2 ± 116.9	311.9 ± 246.9	186.1 ± 82.6	249.0 ± 183.2	373.8 ± 213.8
	All enrichment items		432.3 ± 193.3	536.8 ± 249.6	484.6 ± 224.7	313.5 ± 170.8	325.9 ± 184.9	319.7 ± 175.2	390.4 ± 212.6
Number of interactions (N)	Dry hide		3.37 ± 0.76	5.30 ± 1.23	4.33 ± 1.39	7.96 ± 2.28	7.19 ± 2.18	7.58 ± 2.10	6.19 ± 2.43
	Feeding		3.30 ± 0.93	3.22 ± 2.19	3.26 ± 1.50	3.68 ± 1.72	5.92 ± 1.59	4.80 ± 1.94	4.14 ± 1.88
	New object		1.70 ± 0.39	2.30 ± 0.94	2.00 ± 0.72	1.90 ± 1.37	1.67 ± 0.65	1.78 ± 1.00	1.88 ± 0.87
	Wet hide		2.67 ± 0.51	3.04 ± 0.90	2.85 ± 0.69	3.32 ± 1.88	4.56 ± 0.83	3.94 ± 1.50	3.47 ± 1.30
	All enrichment items		2.76 ± 0.91	3.46 ± 1.68	3.11 ± 1.37	4.22 ± 2.86	4.83 ± 2.48	4.52 ± 2.65	3.92 ± 2.29
Total time of engagement with enrichment (s)	Dry hide		736.4 ± 164.1	814.8 ± 166.9	775.6 ± 154.1	987.6 ± 196.6	1044.5 ± 198.6	1016.1 ± 185.4	913.0 ± 207.1
	Feeding		143.2 ± 30.6	169.9 ± 48.2	156.5 ± 38.9	192.1 ± 37.7	229.1 ± 68.9	210.6 ± 55.1	187.4 ± 54.7
	New object		51.5 ± 6.2	97.0 ± 43.1	74.3 ± 37.2	89.3 ± 102.1	71.1 ± 27.3	80.2 ± 69.9	77.7 ± 56.3
	Wet hide		1039.6 ± 64.2	974.3 ± 116.5	1006.9 ± 91.4	1076.5 ± 198.3	1208.6 ± 216.5	1142.6 ± 204.7	1084.4 ± 175.0
	All enrichment items		492.7 ± 435.9	514.0 ± 412.9	503.3 ± 415.3	586.4 ± 482.0	638.3 ± 528.7	612.4 ± 498.3	565.6 ± 463.8

## Data Availability

The data presented in this study are available on request from the corresponding author.

## Supplementary Materials

The following supporting information can be downloaded at https://www.mdpi.com/article/10.3390/ani13061111/s1: Table S1. Behavior types observed during the study.
