# Supplementary material for: The Effect of Enrichment on Leopard Geckos (Eublepharis macularius) Housed in Two Different Maintenance Systems (Rack System vs. Terrarium)
Source: Animals (Basel). 2023 Mar 21;13(6):1111. doi: 10.3390/ani13061111 (PMC10044651; doi:10.3390/ani13061111)
Supplement: Supplementary file 1 [file animals-13-01111-s001.zip › animals-2067347-Supplementary.pdf]

Table S1. Behavior types observed during the study

Type of enrichment	Behavior type	Definition
Wet hide	watch	Observation with the eyesight directed towards the enrichment with the stationary position of the body
	lick	Ejecting and retracting the tongue for contact with the environment and enrichment
	sniff	Direct nasal contact with the enrichment for exposure to the scent
	circle the enrichment	Moving around the enrichment with the body very close to the object; occasional contact by touching it with the tongue
	climb	Climbing onto the enrichment; descending from the enrichment
	rest on the lid	Rest with the whole body on the enrichment with the body in the motionless position
	rest inside	Rest with the whole body inside the enrichment; lying on the substrate
	dig in the substrate	Digging in the substrate while in the middle of the enrichment; occasional contact by touching it with the tongue
Dry hide	watch	Observation with the eyesight directed towards the enrichment with the stationary position of the body
	lick	Ejecting and retracting the tongue for contact with the environment and enrichment
	sniff	Direct nasal contact with the enrichment for exposure to the scent
	circle the enrichment	Moving around the enrichment with the body very close to the object; occasional contact by touching it with the tongue
	climb	Climbing onto the enrichment; descending from the enrichment
	rest on the hide	Rest with the whole body on the enrichment with the body in the motionless position
	rest under the hide	Rest with the whole body underneath the enrichment with the body in the motionless position
	scratch	Intentional use of claws for scratching the enrichment (claw abrasion); rubbing the body against the enrichment
Feeding	watch	Observation with the eyesight directed towards the enrichment with the stationary position of the body
	lick	Ejecting and retracting the tongue for contact with the environment and enrichment
	sniff	Direct nasal contact with the enrichment for exposure to the scent
	climb	Climbing onto the enrichment; descending from enrichment; passing by the enrichment
	hunt	Attempt to hunt insects inside the enrichment; species-specific tail movements
	manipulate	Manipulating (holding) an object with one or more feet
New object	watch	Observation with the eyesight directed towards enrichment with the stationary position of the body
	lick	Ejecting and retracting the tongue for contact with the environment and enrichment
	sniff	Direct nasal contact with the enrichment for exposure to the scent
	climb	Climbing onto the enrichment; descending from the enrichment; passing by the enrichment
	manipulate	Manipulating (holding) an object with one or more feet
